# Human neutrophils communicate remotely via calcium-dependent glutamate-induced glutamate release

**DOI:** 10.1016/j.isci.2023.107236

**Published:** 2023-06-28

**Authors:** Olga Kopach, Sergyi Sylantyev, Lucie Bard, Piotr Michaluk, Janosch P. Heller, Ana Gutierrez del Arroyo, Gareth L. Ackland, Alexander V. Gourine, Dmitri A. Rusakov

**Affiliations:** 1 Queen Square Institute of Neurology, University College London, Queen Square, London WC1N 3BG, UK; 2 Rowett Institute, University of Aberdeen, Ashgrove Road West, Aberdeen AB25 2ZD, UK; 3 Translational Medicine and Therapeutics, Queen Mary University of London, Mile End Road, London E1 4NS, UK; 4 Department of Neuroscience, Physiology and Pharmacology, University College London, Gower Street, London WC1E 6BT, UK; 5 BRAINCITY, Laboratory of Neurobiology, Nencki Institute of Experimental Biology PAS, 3 Pasteur Street, 02-093 Warsaw, Poland; 6 School of Biotechnology, Dublin City University, Dublin 9, Ireland

**Keywords:** biological sciences, cell biology, immunology, molecular biology, neuroscience

## Abstract

Neutrophils are white blood cells that are critical to acute inflammatory and adaptive immune responses. Their swarming-pattern behavior is controlled by multiple cellular cascades involving calcium-dependent release of various signaling molecules. Previous studies have reported that neutrophils express glutamate receptors and can release glutamate but evidence of direct neutrophil-neutrophil communication has been elusive. Here, we hold semi-suspended cultured human neutrophils in patch-clamp whole-cell mode to find that calcium mobilization induced by stimulating one neutrophil can trigger an N-methyl-D-aspartate (NMDA) receptor-driven membrane current and calcium signal in neighboring neutrophils. We employ an enzymatic-based imaging assay to image, in real time, glutamate release from neutrophils induced by glutamate released from their neighbors. These observations provide direct evidence for a positive-feedback inter-neutrophil communication that could contribute to mechanisms regulating communal neutrophil behavior.

## Highlights

• Human neutrophils operate Ca^2+^ permeable NMDA receptors
• Stimulating one neutrophil activates NMDAR-dependent Ca^2+^ entry in neighbors
• Neutrophils release glutamate inducing glutamate release from other neutrophils

## Graphical abstract

**Figure:**
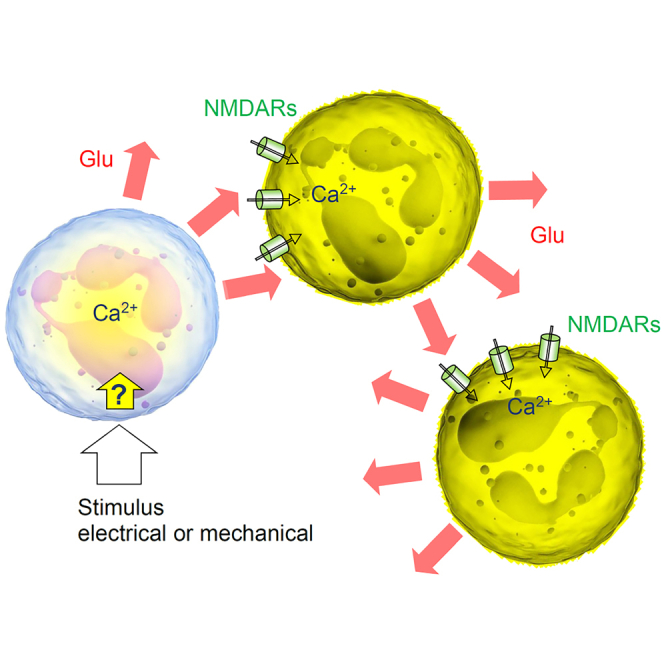

## Introduction

Neutrophils provide the first line of defense against pathogens during acute inflammation.^1^^,^^2^^,^^3^ In humans, a significant decrease in neutrophil numbers could lead to severe immunodeficiency or death. These polymorphonuclear leukocytes mobilize to the site of inflammation and engage several cell-killing mechanisms to clear the infection.^4^^,^^5^ Cellular mechanisms underpinning communication of neutrophils with other actors of a pathological immune response, such as platelets, T cells, or infectious agents, have been intensely studied.^6^^,^^7^^,^^8^

Nonetheless important is the rapidly emerging knowledge of intercellular communication among neutrophils themselves. Cooperative, swarm-like migration patterns of neutrophils have been considered an essential process in their tissue response.^9^^,^^10^ The underpinning molecular mechanisms involve the lipid LTB4 and integrins, the release of signaling molecules such as ATP,^11^ and the action of connexins accompanied by cooperative calcium alarm signals.^12^ Ca^2+^ signaling has long been considered key to the physiological functions of neutrophils,^13^ which are equipped with a variety of membrane receptors, including GPCRs, FcRs, and integrins, capable of mediating Ca^2+^ messages.^14^^,^^15^ Ca^2+^-dependent release of chemo-attractants enables self-sustained paracrine signaling, thus providing positive-feedback amplification that drives self-organized neutrophil ensemble behavior.^16^^,^^17^ Generated in a small group of clustering neutrophils, the molecular signal thus triggers what could be a chain-reaction mechanism communicating with more distant cells attracting them for further swarm growth.^17^ However, direct evidence for regenerative-type neutrophil-neutrophil communication at the cellular level has been scarce.

Previous work has shown that neutrophils express ionotropic glutamate receptors of NMDA type (NMDA receptors, NMDARs) and can secrete glutamate and the NMDAR co-agonist D-serine.^18^^,^^19^ Because NMDAR activation can generate major Ca^2+^ influx and thus engage release machinery in the host cell, we thought it was important to understand whether glutamate-induced glutamate release could contribute to the positive-feedback signal amplification among neutrophil ensembles. To explore this, we engaged single-cell neuroscience techniques that we previously established while exploring signal exchange among central neurons and astrocytes.^20^^,^^21^

## Results

### Neutrophils operate NMDA receptor controlled Ca^2+^ entry

**Figure 1:**
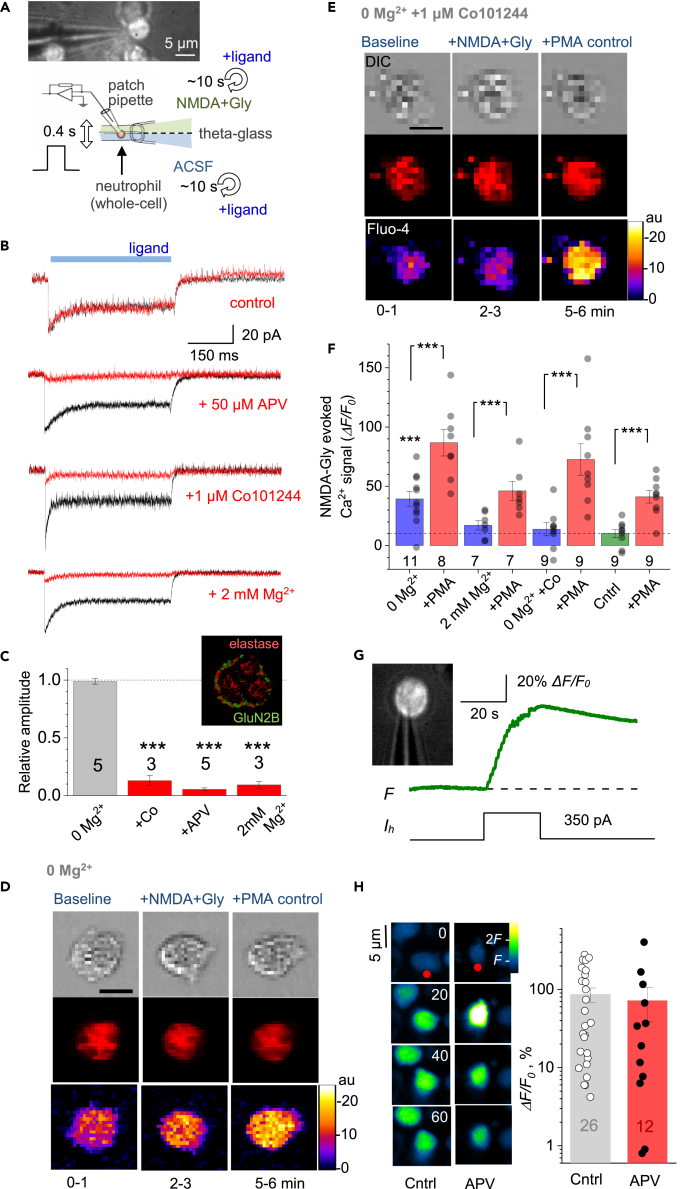
NMDA receptor-mediated Ca^2+^ entry in human neutrophils (A) Diagram depicting probing of NMDARs with a rapid-exchange system [28]: a neutrophil (held in whole-cell) is stimulated pharmacologically by applying different solutions through two channels of ϴ-glass pipette (tip diameter ∼200 μm) mounted on a piezo-drive to enable the ultra-fast delivery (<1 ms resolution; solutions in ϴ glass exchanged within 10 s using a rapid multi-channel perfusion system); see Videos S1 and S2 for live videos of neutrophils before and during patching. (B) Representative whole-cell currents (patch pipette 4–7 MΩ, 1–2 μM tip) recorded in human neutrophils in response to locally applied NMDA (1 mM) and glycine (1 mM), in zero Mg^2+^, 2 mM Mg^2+^, and in the presence of NMDAR antagonists APV (50 μM) and Co101244 (1 μM) in zero Mg^2+^ (control), as indicated; same-cell pharmacological manipulations applied at ∼20 s intervals. (C) Summary of experiments shown in (B); mean ± SEM (amplitude over the 300–500 ms pulse segment), normalized to control (sample size shown); ∗∗∗p < 0.01. Inset, super-resolution dSTORM image of a neutrophil shown with chromatically separated GluN2B and elastase single-molecule labels as indicated; see Figure S1A for further detail and illustrations. (D) Characteristic images of a neutrophil (gray DIC image, top raw) loaded with CellTracker Red (red channel, middle) and Fluo 4-AM (green, bottom), in baseline conditions, after bath application of NMDA (100 μM, 2-3-min duration) and glycine (50 μM), and after adding PMA (1 μM) for Ca^2+^ homeostasis control, as indicated; experimental timing as shown; false color scale: relative intensity, arbitrary units (au); pixel size ∼120 nm (near diffraction limit). (E) Experiment as in (D), but in the presence of the selective GluN2B-containing NMDAR antagonist Co101244 (1 μM); other notations as in (D). (F) Statistical summary of experiments shown in (D and E). Average Ca^2+^ responses (mean ± SEM) of individual neutrophils, first to NMDA+Gly application (blue) and next to PMA (red) application, in either of the three solutions: 0 Mg^2+^ (as in D), 2 mM Mg^2+^, and 0 Mg^2+^ with Co101244 (as in E), as indicated; no NMDA+Gly was applied to control sample (Cntrl, green; 0 Mg^2+^), to evaluate an experiment-wise drift in Ca^2+^-dependent fluorescence at the time of NMDA+Gly response measurement in other groups (dotted line); numbers, sample size (in 0 Mg^2+^ group, 3 cells were lost at the PMA stage); ∗∗∗p < 0.001 (paired *t* test; two-sample *t* test for 0 Mg^2+^ group). (G) One-cell example (neutrophil held in whole-cell, patch pipette is seen) showing that depolarization current induces prominent Ca^2+^ mobilization; inset, DIC+Fluo-4 AM image; traces: *F*, Fluo-4 fluorescence signal; *I*_h_, holding current. (H) Summary of experiments shown in (G). *Left*: Snapshots (Fluo-4 channel) of the stimulated cell (red dot; a neighboring neutrophil can be seen), in control conditions (Cntrl) and in the presence of APV, as indicated, at the time points as indicated (seconds) with respect to the stimulus onset. Note that in control conditions, but not under APV, the neighboring cell responds with a Ca^2+^ rise. *Right*: Summary of depolarization-induced [Ca^2+^]_in_ rises in the patched cell, represented by the Fluo-4 *ΔF/F*_0_ signal; dots, individual cells; bars: mean ± SEM; sample size shown.

We used freshly isolated human neutrophils semi-suspended in a culture preparation that allows for some natural morphological plasticity of these cells (STAR Methods, Video S1). First, to probe directly the function of NMDARs expressed in neutrophils,^19^ we held individual cells in whole-cell mode (current clamp; initially in 0-Mg^2+^ solution). A 1-s pulse of NMDA (100 μM) and the NMDAR co-agonist glycine (1 mM) applied using a piezo-driven theta-glass rapid-solution-exchange system (Figure 1A)^22^ evoked inward current (Figure 1B, top); it was blocked in the same cell either by extracellular Mg^2+^ (2 mM), by the specific NMDAR antagonist 2-amino-5-phosphonovaleric acid (APV, 50 μM), or by the selective antagonist of GluN2B-containing NMDARs Co101244 (1 μM) applied consecutively every 10–20 s through a multi-channel system (Figures 1B and 1C).

Video S1. Examples of live neutrophils under the microscope in differential interference contrast, related to Fig. 1

In separate experiments, we incubated neutrophils with the Ca^2+^ indicator Fluo-4 and morphological tracer CellTracker Red (STAR Methods), to monitor their Ca^2+^ level at each experimental epoch (after adjusting the focal position under two-photon excitation). The agonist stimulus led to a prominent Ca^2+^ rise in control conditions (Figure 1D), but was blocked when 2 mM Mg^2+^ or Co101244 were present in the medium from the start (Figures 1E and 1F). Toward the end of such tests, we confirmed the neutrophil Ca^2+^ signaling capacity (to avoid false negatives) by applying the protein kinase C activator phorbol myristate acetate (PMA, 1 μM), which triggered a further Ca^2+^ elevation in each such case (Figures 1E and 1F).

These observations provide direct evidence for functional NMDARs in neutrophils, predominantly GluN2B subtype, revealing an important mechanism of Ca^2+^ mobilization in these cells. We also used super-resolution microscopy (dSTORM) to further confirm significant expression of GluN2 subunits in human neutrophils (Figure S1). However, neutrophil depolarization could also produce a robust Ca^2+^ rise that was insensitive to APV (Figure 1H), pointing to the additional, voltage-dependent and NMDAR-independent routes of Ca^2+^ entry, such as Calcium release activated (CRAC) channels^15^ or mechanoreceptor transient receptor potential melastatin (TRPM) channels (^15^^,^^23^^,^^24^ and references therein).

### Stimulating one neutrophil activates NMDARs in neighbors

**Figure 2:**
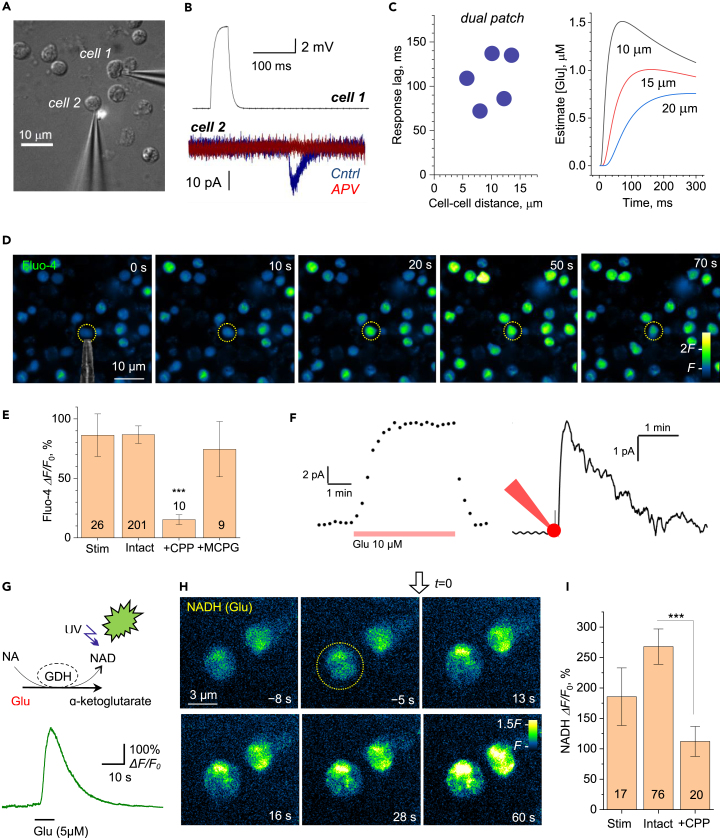
Stimulation of one neutrophil elicits NMDAR-mediated currents, Ca^2+^ rise, and glutamate release in neighboring neutrophils (A) Illustration, dual whole-cell recordings from two human neutrophils (cell 1 and cell 2) semi-suspended in culture; patch-pipette tips can be seen; some neutrophils lie destroyed after unsuccessful dual-patch attempts. (B) Example traces from experiments in (A). A 50 ms current pulse applied in cell 1 (upper trace; current clamp) triggers an inward current deflection in cell 2 (voltage clamp), which is blocked by 50 μM APV; bath medium contains 1 mM glycine. (C) Summary of experiments shown in (A and B) for the five recorded neutrophil pairs (*left*), and theoretical estimates of the glutamate concentration time course at three different distances, as indicated, from a small source neutrophil (*right*); see supplemental methods for detail. (D) Single-cell induced spread of Ca^2+^ signals among neutrophils. Time-lapse sequence (green Fluo-4 channel) in a semi-suspension of human neutrophils; dotted circle, neutrophil held in whole-cell mode (patch pipette fragment from DIC image shown) enabling one-cell electrical stimulation; electric stimulus (50 pA current) is applied at time zero to the patched cell; false color scale of Fluo-4 fluorescence *F*, as indicated; see Figure S1 for characteristic traces and Video S3 for the recorded image sequence. (E) Statistical summary of experiments in (D): amplitude of Fluo-4 fluorescence response (peak response; mean ± SEM) at the stimulated cell (Stim), neighboring intact neutrophils (Intact), and in the presence of the NMDAR blocker 3-((+)-2-carboxypiperazin-4-yl)propyl-l-phosphonic acid (CPP, 10 μM; +CPP) or the wide-range metabotropic glutamate receptor blocker alpha-methyl-4-carboxyphenylglycine (MCPG, 400 μM, + MCPG); numbers inside bars, sample size; ∗∗∗, p < 0.001 (two-sample *t* test, with respect to the three other sample means); see Videos S4 and S5 for recoded examples in CPP and MCPG samples. Note that peak responses were detected in different cells at different times post-stimulus. The data represent 26 independent tests (coverslips) from 10 individual blood cell preparations. (F) *Left*: Glutamate biosensor sensitivity test: a characteristic response to a 10 μM glutamate application, as indicated; dots, sensor record taken every 15 s. *Right*: A glutamate biosensor record during 10 s whole-cell current injection (within 1 min from the sensor recording onset; indicated by red cell-patch diagram) in an individual neutrophil held in whole-cell, as in (D), with sensor recording continuing for approximately 5 min (see Figures S2B–S2D for further detail). (G) *Top*: Schematic of the glutamate imaging method; A fluorescent enzymatic-based assay involves conversion of β-nicotinamide adenine dinucleotide (NAD) by L-glutamic dehydrogenase (GDH) (in the presence of glutamate) to NADH that fluoresces upon UV light excitation. *Bottom*: Assay sensitivity text; a fluorescence response to a pressure pulse of 5 μM glutamate (∼1 μm pipette tip; fluorescence intensity average over a 10 μm × 10 μm area around the tip, to roughly represent the vicinity of an individual neutrophil); see Figure S2 for fluorescent time-lapse snapshots. (H) Time-lapse series of fluorescence images (NADH channel) depicting a prominent rise in extracellular glutamate near the two neutrophils upon mechanical stimulation (light touch by a 1 μm micropipette tip) of one cell (dotted circle) at *t* = 0 s, as indicated; false color scale; note that the glutamate concentration signal drops sharply away from the cell surfaces reflecting rapid dilution in the bath medium. (I) Statistical summary of experiments in (H): amplitude of glutamate-sensitive NADH fluorescence response (mean ± SEM) at the stimulated cell (Stim), neighboring intact neutrophils (Intact), and in the presence of the NMDAR blocker CPP (10 μM; +CPP); numbers inside bars, sample size; ∗∗∗, p < 0.001 (two-sample *t* test). The data represent 17 independent tests (coverslips) from 3 individual blood cell preparations; see Videos S6 and S7 for the illustration of recordings in Intact and CPP samples.

Second, to obtain further direct evidence for remote neutrophil-neutrophil interaction, we held two neutrophils positioned 5–15 μm apart, in whole-cell mode (Figure 2A): these experiments were highly challenging as neutrophils tried to “escape” the patching pipette (Video S2) and stayed in whole-cell mode for several minutes only. Once in a dual-patch configuration, we applied a depolarizing stimulus to one cell to trigger its intracellular Ca^2+^ rise (as Figure 1G). Remarkably, this evoked an APV-sensitive inward current in the other patched cell (Figure 2B). The response occurred 70–140 ms after the stimulus (Figure 2C, left). With glutamate diffusivity of ∼0.7 μm^2^/ms,^25^ diffusion theory indeed suggests a 50–150 ms post-release lag, depending on the separating distance, before glutamate concentration reaches its peak (Figure 2C, right). Intriguingly, for the glutamate concentration to peak at the minimum level sufficient for NMDAR activation (0.5–1 μM) 5–10 μm away, the neutrophil should generate a 5–10 μM glutamate source emanating from its surface (see STAR Methods).

Video S2. Example of a neutrophil moving around the tip of a patching pipette during the whole-cell attempt In this video, the glass patch pipette tip moves in front of the objective trying to catch up with a neutrophil; this introduces various distortions in the differential interference contrast image, which are hard to avoid related to Fig. 1

To understand whether this signal exchange triggers intracellular Ca^2+^ mobilization, we monitored intracellular Ca^2+^ in pairs or groups of neutrophils. The depolarizing stimulus applied to one cell triggered a transient Ca^2+^ rise in its neighbors (Figure 2D; characteristic traces shown in Figure S2A and Video S3). This rise was blocked by the specific NMDAR antagonist 3-((+)-2-carboxypiperazin-4-yl)propyl-l-phosphonic acid (CPP) but not by the broad-range metabotropic receptor antagonist alpha-methyl-4-carboxyphenylglycine (MCPG), although in the latter case the cell Ca^2+^ response was significantly scattered (Figure 2E, Videos S4, and S5).

Video S3. Example of Ca2+ signals in multiple neutrophils induced by stimulation of one cell Red dot indicates stimulation period at the stimulated cell related to Fig. 2D

Video S4. Example of variable Ca2+ signals in several neutrophils induced by stimulation of one neighboring cell, in the presence of the NMDAR blocker CPP Red dot indicates stimulus onset at the stimulated cell. Fluo-4 fluorescent channel, false color scale (characteristic scale bar in Fig. 2D) related to Fig. 2E

Video S5. Example of Ca2+ signals, or the lack of them, in several neutrophils induced by stimulation of one neighboring cell, in the presence of the mGluR blocker MVCPG Red dot indicates stimulus onset at the stimulated cell. Fluo-4 fluorescent channel, false color scale (characteristic scale bar in Fig. 2D) related to Fig. 2E

### Glutamate-induced glutamate release from neutrophils

Finally, we sought to determine whether glutamate released from one neutrophil prompts glutamate release from its neighbors. We first used a glutamate-specific biosensor^26^ (Figure 2F, left) to show that a depolarizing stimulus at one cell produces a micromolar-range glutamate response at 5–15 μm from it (Figure 2F, right).^18^^,^^19^ Next, to visualize directly the fate of glutamate during neutrophil signaling, we employed an established enzymatic assay for extracellular glutamate imaging^27^^,^^28^ (Figure 2G, top). While the sensitivity chart for the assay in steady-state (equilibrated) conditions of glutamate application has been reported,^29^ presently we deal with transient point sources of glutamate, with its concentration dropping sharply away from the release site. We, therefore, confirmed the robust sensitivity of the assay by imaging its fluorescence time course in the vicinity of a patch-pipette tip following a brief pressure puff of glutamate (5 μM at the source; Figure 2G, bottom; Figures S2B–S2D).

We next monitored pairs or small groups of neutrophils using the enzymatic assay and found that even a brief stimulation of one neutrophil (light touch of a patch-pipette) induced pronounced glutamate rises, both at the stimulated and the neighboring cell (Figure 2H, Video S6). The level of fluorescence response in simulated or neighboring neutrophils (200–300% *ΔF/F*_*0*_, Figure 2I) was comparable with that in the pipette-puff test in the vicinity of a 5 μM glutamate source (Figure 2G, bottom; Figure S2D). This may suggest that the endogenously released glutamate could indeed reach a local level of several micromolar, near the surface of both stimulated (n = 17) and remote cell (n = 76; p = 0.362; Figure 2I). In the case when swarming neutrophils are in relative proximity of one another, this level appears not far from the concentration range of ∼8 μM considered optimal for neutrophil chemotaxis and cytoskeleton polarization.^30^ Intriguingly, blocking NMDARs suppressed glutamate-induced glutamate release incompletely, with some signaling remaining (Figure 2I, Video S7), although the latter could be partly the effect of the background glutamate diffusing from the stimulated cell.

Video S6. Example of glutamate-dependent fluorescence signals in several neutrophils induced by stimulation of one neighboring cell, in control conditions Red dot indicates stimulus onset at the stimulated cell. Fluo-4 fluorescent channel, false color scale (characteristic scale bar in Fig. 2G) related to Fig. 2G

Video S7. Example of glutamate-dependent fluorescence signals in several neutrophils induced by stimulation of one neighboring cell, in the presence of the NMDAR blocker CPP Red dot indicates stimulus onset at the stimulated cell. Fluo-4 fluorescent channel, false color scale (characteristic scale bar in Fig. 2G) related to Fig. 2G

## Discussion

### Glutamatergic neutrophil-neutrophil signaling

Cooperative neutrophil behavior, such as swarming, appears to rely on Ca^2+^-induced release of chemo-attractants, with the paracrine-autocrine actions providing positive-feedback signal amplification.^16^^,^^17^^,^^31^ This involves release of various signaling molecules that target their receptors on the neutrophil surface.^11^^,^^12^ Previous studies have indicated that neutrophils express NMDARs and can release glutamate.^18^^,^^19^ We, therefore, hypothesized that glutamate-induced glutamate release could contribute to the self-propagating, communal molecular signaling among neutrophils.

Our experiments demonstrated direct signal exchange among human neutrophils, which enacts through Ca^2+^-dependent glutamate release from one or more cells, activating NMDAR-mediated Ca^2+^ entry and glutamate release in the neighboring neutrophils. To image glutamate-induced glutamate release in a direct manner, we employed an enzymatic assay developed earlier^32^ and validated in astroglial cultures.^28^^,^^29^ The assay solution may partly buffer the freely diffusing glutamate, depending on its spatiotemporal dynamics in relation to the enzymatic reaction kinetics. However, the fact that we could still see remote, NMDAR-dependent glutamate signals in the assay suggests that our observations could actually underestimate such signals. Given the strong glutamate release potential of individual neutrophils, these cells must also have a powerful mechanism of glutamate uptake, as reported earlier,^33^ particularly because an excess of extracellular glutamate could hamper neutrophil cell-killing abilities.^34^ Although we detected no significant role of metabotropic glutamate receptors in the Ca^2+^ signal exchange among neutrophils, these receptors could still contribute to their migratory behavior *in vivo*.^35^^,^^36^ Intriguingly, recent data point to a metabotropic, Ca^2+^-entry independent action of NMDARs interacting directly with mGluRs in neutrophils.^37^ Thus, we cannot rule out residual contribution of native mGluRs to Ca^2+^ signals in response to glutamate: the wide scatter of MCPG-dependent data (Figure 2E, Video S5) suggests that the mGluR blockade could decrease Ca^2+^ response in some cells.

Similarly, evidence has been emerging for the regulatory roles of mechano-sensing TRP channels in neutrophils,^38^ in particular their potential contribution to neutrophil chemotaxis *in vivo*.^39^ While the role of TRP receptors is outside the scope of the present study, it is possible that both patch-clamp interference and mechanical stimulation of individual neutrophils involves TRP activation leading to an NMDAR-independent Ca^2+^ mobilization, as reported here (Figure 1H). Notably, our key tests were carried out in low Mg^2+^ because NMDARs are subject to the Mg^2+^ block unless the cell membrane is depolarized.^40^ It is, therefore, a plausible assumption that such depolarization *in vivo* might be prompted by TRP activation. Alternatively, the membrane potential of neutrophils, which is maintained mainly by their Na^+^/K^+^ pumps,^41^ can undergo drastic depolarization during neutrophil activation,^42^ thus exposing NMDARs to direct activation by glutamate. The latter suggests that our observations might be particularly relevant to neutrophil behavior under activation.

### Indicators of regenerative signal propagation

Overall, our observations point to glutamate-induced glutamate release as a mechanism that could provide regenerative, self-maintained signal propagation through the neutrophil ensemble. At the same time, our focus on the glutamate-release NMDAR-dependent neutrophil signaling does not exclude, but rather complements, other signaling mechanisms associated with self-organization and pattern behavior of these cells *in vivo*. Swarming and dynamic clustering have been considered an essential process in their tissue response.^9^^,^^10^ In this respect, recent observations of neutrophil behavior *in vivo* report a cooperative intracellular Ca^2+^ mobilization, or “calcium alarm” signal upon contact of neutrophils with their target.^12^^,^^43^ Whether such signals are accompanied by the chain reaction of glutamate-induced glutamate release as reported here remains an intriguing question. However, glutamate release-triggered NMDAR-dependent signaling has recently been implicated in the intercellular communication within the neurovascular unit^44^ and in neuroendocrine tumor cells,^45^ lending support to the emerging view of the important role of this glutamate-dependent mode of intercellular communication in peripheral organs.^46^

### Limitations of the study

While the present results provide strong support for the latter hypothesis, our approach has important limitations. Firstly, having neutrophils semi-suspended in culture is important for patch-clamp recordings and reliable Ca^2+^ and glutamate imaging, but it restricts free cell movement, thus depriving us of exploring pattern behaviors of the cell ensemble. We also noted that a small fraction of neutrophils in our preparations displayed spontaneous Ca^2+^ discharges. In the majority of cells, the Ca^2+^ “wave” was triggered by the stimulus, but for the small minority, we could not determine whether their activity was spontaneous or stimulus-triggered. That the spontaneous Ca^2+^ activity in some cells did not trigger Ca^2+^ waves, or that some cells did not respond to the stimulus, suggests, as the most parsimonious explanation, that some neutrophils in our experiments did not possess the mechanisms of Ca^2+^-dependent glutamate-induced glutamate release. Secondly, to ensure consistency of our experiments, which requires reproducible conditions of cell stimulation across varied protocols, we employed well-controlled electrical or light-touch mechanical stimuli of individual neutrophils. Clearly, it would not be possible to avoid “touch” when using patch-clamp, whereas using “touch” alone was less perturbing but more difficult to interpret. We could, therefore, only assume that such stimulation could represent, at least partly, real-life scenarios in which neutrophils face foreign bodies causing an inflammatory response.

## STAR★Methods

### Key resources table

**Table:** 

REAGENT or RESOURCE	SOURCE	IDENTIFIER
**Biological samples**		

Blood cells	healthy volunteers	anonymous

**Chemicals, peptides, and recombinant proteins**		

CO-101244	Tocris Bioscience	Cat # 2456
Phorbol 12-myristate 13-acetate	Tocris Bioscience	Cat # 1201
NMDA	Tocris Bioscience	Cat # 0114
(R)-CPP	Tocris Bioscience	Cat # 0247
Minimal Essential Media	Thermo Fisher	Cat # 21090-022
HBSS	Thermo Fisher	Cat # 14185-045
B27	Thermo Fisher	Cat # 12587010
HEPES solution	Merck	Cat #H0887-20ML
Minimal Essential Media	Thermo Fisher	Cat # 21090-022
Alexa Fluor 594 hydrazide	Thermo Fisher	Cat. #A10438
Fluo-4/AM	Thermo Fisher	Cat. #F-14201
Glycine	Tocris Bioscience	Cat. #0219
CPP	Sigma-Aldrich	Cas #:100828-16-8
MCPG	Tocris Bioscience	Cat. #0336
L-Glutamic dehydrogenase	Sigma-Aldrich	Cat. # 9029-12-3
β-Nicotinamide adenine dinucleotide hydrate	Sigma-Aldrich	N7004-1G
Cell tracker red	Thermo Fisher	C34552
Pluronic™ F-127	Thermo Fisher	Cat. #P-6866
Histopaque®-1077	Sigma-Aldrich	Cat. #1077
HEPES	Sigma-Aldrich	Cas #: 7365 45 9
HBSS (-Mg^2+^/−Ca^2+^)	Thermo Fisher	Cat. #14025092
Poly-D-Lysine	Thermo Fisher	Cat. # A3890401
Dextran	Sigma-Aldrich	Cas #: 9004-54-0
PBS	Sigma-Aldrich	Cat. #P4417
(+)-MK-801 hydrogen maleate	Sigma Aldrich	Cat. #M107
L-Glutamic acid monosodium salt monohydrate	Sigma-Aldrich	49621
D-APV	Abcam	Cat. #ab120003
DL-AP5	Bio-Techne Ltd	Cat. # 0105
NBQX disodium salt	Abcam	Cat. #ab120046
Pertussis toxin	Tocris Bioscience	Cat # 3097

**Software and algorithms**		

pClamp 10	Molecular Devices	RRID:SCR_011323
ImageJ Fiji	NIH	RRID:SCR_003070
OriginPro	OriginLab Inc.	RRID:SCR_014212

**Other**		

Digidata 1440A digitizer	Molecular Devices	RRID:SCR_021038
MultiClamp 700B amplifier	Molecular Devices	RRID:SCR_018455
Olympus Fluoview1000	Olympus	RRID:SCR_014215
Olympus BX51 Fluorescence Microscope	Olympus	RRID:SCR_018949
Micromanager4.1	NIH	https://micro-manager.org/
Evolve 512 EMCCD camera	Photometrics	https://www.photometrics.com/products/emccd-family

### Resource availability

#### Lead contact

Further information and requests for resources and reagents should be directed to and will be fulfilled by the lead contact, Dmitri Rusakov (d.rusakov@ucl.ac.uk).

#### Materials availability

• This study did not generate new unique reagents.

### Experimental model and study participant details

#### Preparation of human neutrophils

Neutrophils were isolated from blood samples obtained by venepuncture from anonymized healthy volunteers, males and females, British adults who self-reported their race as “White”, ages from 30 to 45, according to protocols approved by the UCL Research Ethics Committee (UCL Queen Square Institute of Neurology, London, UK), with the informed consent of participants.

For the isolation of neutrophils, we used a method of dextran sedimentation and differential centrifugation through a Ficoll-Hypaque density gradient as described in detail elsewhere.^47^ Briefly, a sample of the whole blood was suspended in sodium citrate solution, and a suspension of neutrophils was obtained by sedimentation in 2% dextran (Sigma, UK) in 0.9% NaCl for 45–60 min at room temperature. The neutrophil-enriched upper layers of the suspension were collected and centrifuged (1150 rpm, 10 min at 4°C). Residual erythrocytes were removed by hypotonic lysis, and the obtained suspension was further centrifuged (1300 rpm, 6 min at 4°C); the pellet was further re-suspended in PBS and purified by gradient centrifugation over Ficoll-Hypaque (Sigma, cat. 1077; 1500 rpm, 30 min at 4°C). The resulting pellet containing neutrophils was finally re-suspended in HBSS (0-Mg^2+^/0-Ca^2+^); cells were plated on coverslips coated with poly-DL-Lysine (1 mg/mL) and laminine (20 μg/mL) and were maintained until used (37°C, 5% CO_2_). With this protocol, neutrophils remained lightly attached to the coverslip, forming a semi-suspension: this partly restricted their movement while allowing for patch-clamp and fluorescence imaging experiments in individual cells. At the same time, the cells were not flattened by strong adhesion and showed considerable morphological plasticity on the microscopic scale. We thus identified healthy cells by their normal morphology, including pseudopodia motility, stimulus evoked transient Ca^2+^ responses, normal NMDAR-dependent activity, and evoked glutamate release. The cultures contained a significant proportion of apparently healthy, stimulation-responsive neutrophils for up to 24 h, but the present data were collected within the fist several hours post-isolation.

### Method details

#### Patch-clamp electrophysiology

Visualized patch-clamp recordings from the neutrophils were performed using a Multipatch 700B amplifier controlled by pClamp 10.2 software package (Molecular Devices, USA). For the recordings, cells on a glass coverslip were placed in a recording chamber mounted on the stage of an Olympus BX51WI upright microscope (Olympus, Japan). The perfusion medium contained (mM): 119 NaCl, 2.5 KCl, 1.3 MgSO_4_, 2.5 CaCl_2_, 26.2 NaHCO_3_, 1 NaH_2_PO_4_, 10 glucose and was continuously bubbled with 95% O_2_ and 5% CO_2_ (pH 7.4; 290–298 mOsm).). For the recordings of the NMDAR-mediated currents, the medium was changed for a modified zero-Mg^2+^ solution that contained (mM) 119 NaCl, 2.5 KCl, 2.5 CaCl2, 26.2 NaHCO3, 1 NaH2PO4, 23 glucose (pH 7.4; 290–298 mOsm). The NMDAR-mediated currents were measured in voltage-clamp mode, V_hold_ = −70 mV at 33–34°C. Signals were digitized at 10 kHz. Patch pipettes were pulled from borosilicate glass and fire-polished to 4–7 MΩ (1.5–3 μm tip). The intracellular pipette solution for voltage-clamp experiments contained (mM): 120.5 CsCl, 10 KOH-HEPES, 2 EGTA, 8 NaCl, 5 QX-314 Br^−^, 2 Mg-ATP, 0.3 Na-GTP, 10 Na-phosphocreatine, pH and osmolarity adjusted to 7.2 and 295 mOsm, respectively. For whole-cell experiments in current mode, an internal solution contained (in mM) 135 K-methylsulphonate, 4 MgCl_2_, 10 Tris-phosphocreatine, 10 HEPES, 4 MgATP, 0.4 GTP-Na (pH 7.2, osmolarity 290 mOsm, pipette resistance 4–7 MΩ). Stimulating protocol consisted of a series of 500-ms depolarizing rectangular current pulses injected into a neutrophil with an increment of 20–30 pA (input current was injected up to 300–350 pA).

The previously established fast-application system^22^ included a theta-glass application pipette with ∼200 μm tip diameter attached to the PL127.11 piezo actuator driven by the E−650.00 LVPZT amplifier (both from PI Electronics). Routinely, one pipette channel was filled with the bath solution and the other channel was filled with the bath solution containing 100 μM NMDA and 1 mM glycine, or alternatively with NMDA and glycine, plus NMDAR blockers such as APV or CPP (the latter is considered potent and therefore is more frequently used when the extent of glutamate glutamate transients is not known). The pressure in the application pipette was regulated by a two-channel PDES-02DX pneumatic micro ejector (npi electronic GmbH) using compressed nitrogen. To test the effects of various ligands, the application solutions in both theta-glass pipette channels could be exchanged within 10–12 s during the experiment using dedicated pressurized micro-circuits. NMDAR ligands were applied in 400 ms pulses 5 s apart.

#### Diffusion time course estimates

The glutamate concentration time course *C*(*r*,*t*) at distance *r* from the glutamate-releasing cell was estimated, as a first approximation, using the classical equation for a small source in an infinite volume:

$$C\left(r,t\right)=\frac{C_{0}V_{0}}{{8\left(\pi Dt\right)}^{1/2}}\exp\left(-\frac{r^{2}}{4Dt}\right)$$

where *D* = 0.7 μm^2^/ms is glutamate diffusivity, and *C*_0_ stands for glutamate concentration at *t* = 0 within small source volume V_0_. For the sake of simplicity, in the calculations we assumed that that volume from which glutamate is released can be represented by a 0.5 μm layer around a 6 μm wide spherical neutrophil. To reach the minimum glutamate concentration required to activate NMDARs (0.5–1 μM), this equation predicts *C*_0_ in the range of ∼5 μM (or equivalently, ∼10 μM within a 0.25 μM layer around the neutrophil source).

#### Two-photon excitation (2PE) fluorescence imaging

For live-cell imaging, neutrophils were loaded with a morphological tracer CellTracker Red (5 μM, Invitrogen) and Ca^2+^-indicator Fluo-4/AM (5 μM, Invitrogen) in the presence of Pluronic F-127 (0.02%, Invitrogen) for 10 min at 30⁰C. After incubation with the dyes, cells were washed out for 10 min in a medium containing (mM): 119 NaCl, 2.5 KCl, 1.3 MgSO_4_, 2 CaCl_2_, 26 NaHCO_3_, 1.25 NaH_2_PO_4_, 12 glucose (95% O_2_ and 5% CO_2_; pH 7.4, 290–300 mOsm). Imaging was performed in a medium of the same composition containing either 0 or 2 mM MgSO_4_, as specified, at 30–33⁰C. Imaging was carried out using an Olympus FV-1000MPE system optically linked to a Ti:Sapphire MaiTai femtosecond pulse laser (SpectraPhysics Newport) at λ^2P^_ex_ = 800 nm, with appropriate emission filters (Fluo-4: 515–560 nm band; Cell tracker red: 590–650 nm band), as detailed previously.^48^^,^^49^ For the time-lapse recordings, *z*-stacks of fluorescent images (containing 5–10 cells within the field of view) were collected in a 1-min increment for 2 min before (baseline) and upon application of NMDA (100 μM) and glycine (50 μM) (bath application for the next ∼3 min). At the end of each experiment, the protein kinase C activator phorbol myristate acetate (PMA, 1 μM) was added for 2 min as a functional test. For the analysis, the Fluo-4 signal (*G*, green channel) was normalized to the Cell tracker fluorescence (*R*, red channel), and changes in Ca^2+^ level were first normalized as *ΔG/R* after background subtraction and next presented as *ΔF/F*_*0*_ change in Fluo-4 fluorescence. Only cells displaying a stable baseline and robust (>2-fold) *ΔG/R* increase in response to the PMA test, with no Fluo-4 saturation, were included in the statistics.

#### Fast wide-field fluorescence imaging

Neutrophils were loaded with Fluo-4 a.m. (5 μM, Invitrogen) in the presence of Pluronic F-127 (0.02%, Invitrogen) for 15 min at 37⁰C. After loading, cells were washed out for approximately 30 min in a Ringer solution containing (in mM) 126 NaCl, 3 KCl, 2 MgSO_4_, 2 CaCl_2_, 26 NaHCO_3_, 1.25 NaH_2_PO_4_, 10 glucose, equilibrated with 95% O_2_ and 5% CO_2_ (pH 7.4; 290–300 mOsm). Experiments were performed at 30–33⁰C, using an Olympus BX51WI upright microscope (Olympus, Japan) equipped with a LUMPlanFI/IR 40 × 0.8 objective and an Evolve 512 EMCCD camera (Photometrics). A source of fluorescent light was an X-Cite Intelli lamp (Lumen Dynamics). For the time-lapse recordings, 700 to 3000 images were acquired in the stream-acquisition mode (exposure times from 20 to 100 ms), using MetaFluo (Cairn Research Ltd, UK) or Micromanager 4.1 (freely available ImageJ plugin) software. To collect images at high resolution, various digital zooms were used (100–200 nm per pixel). Changes in the intracellular Ca^2+^ level were expressed as the changes in Fluo-4 fluorescent signal over the baseline (*ΔF/F*_*0*_) after subtraction of background fluorescence and correction for bleaching.

For mechanical stimulation, an individual cell was gently tapped with a glass patch-pipette over the cell surface, under visual control, while monitoring changes in the pipette resistance (confirming the contact with the cell membrane).

#### Glutamate recordings with an electrochemical sensor

For the assessment of changes in the extracellular glutamate level across isolated neutrophils, we used the glutamate-sensitive electrochemical microelectrode biosensors, with a tip of 7-μm in diameter (Sarissa Biomedical Ltd., Coventry, UK). For the recordings, a glutamate biosensor was placed close to the stimulated neutrophil (within 5–15 μm), with a null biosensor located apart. After positioning, the basal current was recorded for a few minutes before (baseline) and upon cell stimulation. Biosensors were calibrated prior to each experiment using 10 μM of glutamate.

#### Glutamate imaging

Fast wide-field imaging was used to visualize glutamate released by individual neutrophils in combination with an enzymatic assay as described earlier,^27^^,^^28^ with some modifications. Before the recordings, isolated neutrophils were perfused with a HEPES-based medium containing (in mM) 135 NaCl, 5 KCl, 2 CaCl_2_, 2 MgCl_2_, 10 HEPES, 10 glucose (pH 7.4; 290–300 mOsm). The bath medium was supplemented with L-glutamic dehydrogenase (GDH, 60 U/ml, Sigma, UK) and β-nicotinamide adenine dinucleotide (NAD, 1 mM). In the presence of glutamate, L-glutamic dehydrogenase (GDH) reduces NAD(+) to NADH, a product that fluoresces when excited with either UV light or in two-photon excitation mode.^29^ The detection of released glutamate thus relies on the NADH-mediated fluorescence following the GDH-catalyzed conversion of NAD to NADH in the presence of glutamate. Imaging was performed in a semi-suspension of isolated neutrophils using an Olympus BX51WI upright microscope (Olympus, Japan) equipped with a LUMPlanFI/IR 40 × 0.8 objective and appropriate emission filters (U-MWU2 filter set, Olympus). Fluorescence was collected with a fast-speed ultrasensitive Evolve 512 EMCCD camera (Photometrics). Glutamate imaging recordings were performed at 30–33⁰C in zero-Mg^2+^ solution, with no perfusion running. Experiments were performed at 30–33⁰C. Changes in the NADH fluorescence were calculated as ΔF/F_0_ after the subtraction of background fluorescence. Background subtraction was performed for the time-lapse sequence, by subtracting the optical image before stimulation throughout *T*-stacks to eliminate basal NADH fluorescence and the contaminating fluorescence, such as autofluorescence of enzymes.

#### Two-colour 3D STORM

For two-colour 3D single-molecule localization microscopy (SMLM) we modified a protocol described by us previously.^21^^,^^50^^,^^51^ Wells of 8-well Chambered Coverglass w/non-removable wells (ThermoFisher Scientific, #155411) were treated with 1M KOH for 15 min and washed with H_2_O. Afterward, wells were coated with poly-DL-Lysine (1 mg/mL) and laminin (20 μg/mL). 100,000 neutrophils were plated into each well and incubated at 37°C, 5% CO_2_ for 1 h.

Cells were fixed using 3.7% PFA in PBS at 37°C for 12 min and washed with PBS three times. Non-reacted aldehydes were quenched in 0.1% NaBH_4_ in PBS for 5 min. Cells were washed once for 30 s and once for 5 min with PBS. Cells were then permeabilized using PBS supplemented with 0.1% Triton X-100 for 10 min. Cells were washed for 30 s in PBS before being blocked in 3% bovine serum albumin (BSA) in PBS for 30 min. This was followed by incubation with primary antibody (see below) in PBS overnight at 4°C. Cells were washed once with PBS for 30 s and trice with PBS for 15 min before being incubated with secondary antibody (see below) in PBS for 1 h and 10 min, washed with PBS once for 30 s and twice for 10 min with PBS and post-fixed with 4% PFA in PBS for 15 min. Lastly, cells were washed with PBS thrice for 10 min and stored at 4°C until being prepared for imaging.

Primary antibodies used: neutrophil elastase (Rabbit, Polyclonal, Synthetic peptide conjugated to KLH derived from within residues 1–100 of Human Neutrophil Elastase, Abcam, ab68672, RRID AB_1658868, dilution 1:500) and NMDA receptor subtype 2B (Mouse, Monoclonal, Rat NMDAR2B residues 892–1051, BD Biosciences, 610416, RRID AB_397796, dilution 1:500). Secondary antibodies used: anti-mouse IgG (Donkey, CF568-conjugated, Biotium, 20105, RRID AB_10557030, dilution 1:500), anti-rabbit IgG (Donkey, Alexa 647-conjugated, Thermo Fisher Scientific, A31573, RRID AB_2536183, dilution: 1:1000).

SMLM images were recorded with a Vutara 350 microscope (Bruker). The targets were imaged using 640 nm (for Alexa 647) or 561 nm (for CF568) excitation lasers and a 405 nm activation laser. We used a photoswitching buffer containing 100 mM cysteamine and oxygen scavengers (glucose oxidase and catalase) (Metcalf et al., 2013). Images were recorded using a 60×-magnification, 1.2-NA water immersion objective (Olympus) and a Flash 4.0 sCMOS camera (Hamatasu) with frame rate at 50 Hz. 5000 frames were acquired per channel. Data were analyzed using the Vutara SRX software (version 6.02.05). Single molecules were identified by their continued emission frame-by-frame after removing the background. Identified particles were then localized in three dimensions by fitting the raw data with a 3D model function, which was obtained from recorded bead datasets.

### Quantification and statistical analysis

The experimental design involved real-time recordings with direct in-situ (same cell) comparison, independent factors, and no longitudinal trials. Shapiro-Wilk tests for normality were routinely run for small samples (this test for the means could be misleading for n > 15–19 due to Central Limit Theorem). Correspondingly, two-tailed paired and unpaired Student’s *t* test, or otherwise non-parametric Mann-Whitney tests were used for statistical analyses. Mean difference was considered significant at the null-hypothesis rejection level of p < 0.05. Statistical summary data are shown as mean ± SEM unless specified otherwise.

## Data Availability

• Data reported in this paper will be shared by the lead contact upon request. • This manuscript contains no original programming code. • Any additional information required to reanalyse the data reported in this paper is available from the lead upon request.
