# Supplementary material for: Human neutrophils communicate remotely via calcium-dependent glutamate-induced glutamate release
Source: iScience. 2023 Jun 28;26(7):107236. doi: 10.1016/j.isci.2023.107236 (PMC10366500; doi:10.1016/j.isci.2023.107236)
Supplement: Document S1. Figures S1 and S2 [file mmc1.pdf]

iScience, Volume *26*

Supplemental information

Human neutrophils communicate remotely via calcium-dependent glutamate-induced glutamate release

Olga Kopach, Sergyi Sylantyev, Lucie Bard, Piotr Michaluk, Janosch P. Heller, Ana Gutierrez del Arroyo, Gareth L. Ackland, Alexander V. Gourine, and Dmitri A. Rusakov

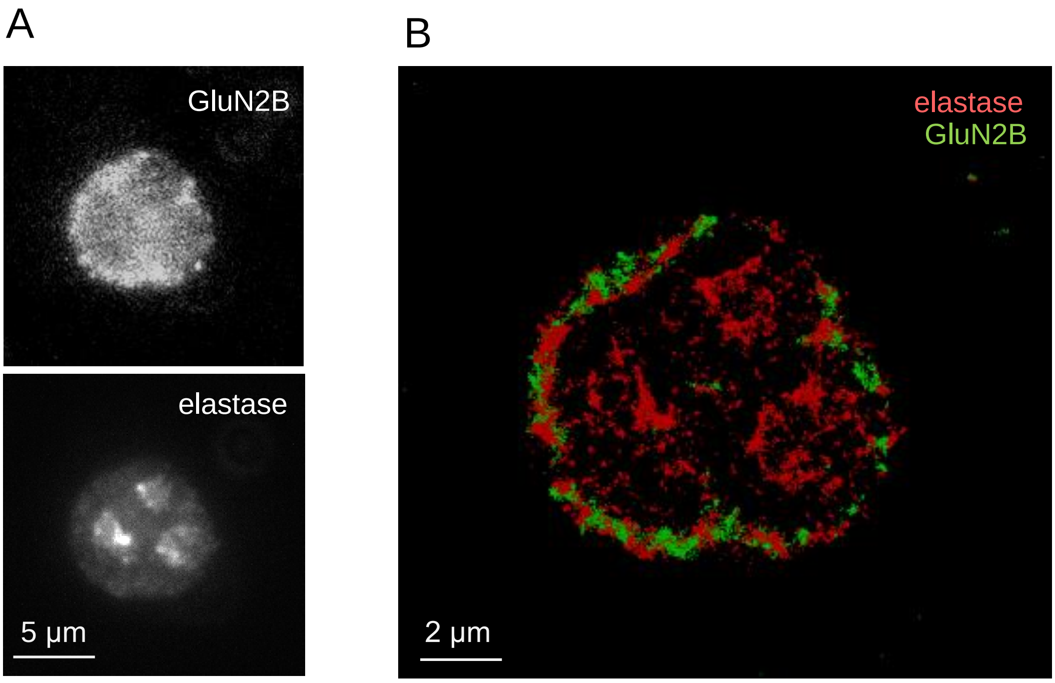

Figure S1. NMDAR GluN2B subunit expression and examples of the Ca^{2+} signal time course in neutrophils following electric stimulation of one cell. Related to Figure 1.

(A) A wide-field fluorescence image of a neutrophil (post-fixation) with antibody labelled NMDAR subunits GluN2B (Alexa 647 channel) and neutrophil elastase (CF568 channel), as indicated.

(B) A dSTORM image of the neutrophil shown in A (z-projection), with chromatically separated GluN2B and elastase single-molecule labels as indicated. See STAR Methods for the labelling protocol and imaging detail.

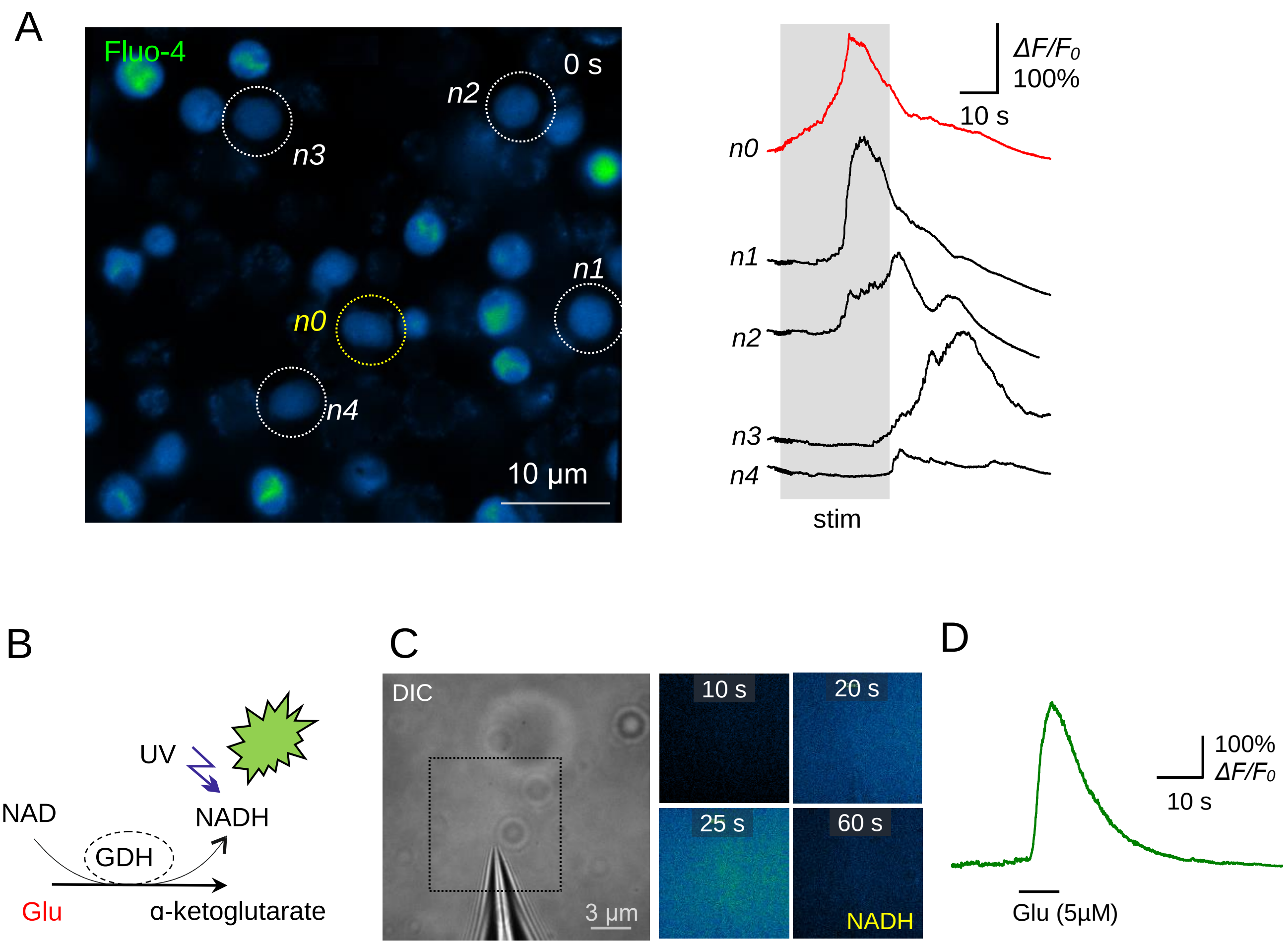

Figure S2. Ca^{2+} signal time course in neutrophils upon one-cell electric stimulation and glutamate sensitivity probing of the enzymatic-based assay. Related to Figure 2.

(A) *Left:* A snapshot of neutrophils incubated in Fluo-4 (epifluorescence channel, false colour sale), as in Fig. 2D at *t* = 0 s, control condition; *n0* denotes patched and stimulated cell. *Right:* fluorescence time course at the stimulated (red line) and four unperturbed neutrophils (*n1*-*n4*) as indicated on the left; shaded area, electric simulation period.

(B) Diagram, fluorescence imaging an enzymatic-based assay: conversion of β-nicotinamide adenine dinucleotide (NAD) by L-glutamic dehydrogenase (GDH) (in the presence of glutamate) to NADH that fluoresces upon UV light excitation.

(C) Glutamate imaging, method validation: DIC image, patch-pipette puffing glutamate (5 μM, 10 s) into the enzymatic-based medium containing GDH (60 U/ml) and NAD (1 mM); colour panels (false colour scale): examples of time-lapse snapshots (time stamp from the recording onset is shown) of NADH fluorescence integrated over the 10 μm x 10 μm ROI; two-photon excitation, λ_x^{2P} = 800 nm.

(D) Time course of NADH-mediated fluorescence in the experiment shown in (B).
